# Support Tools in the Differential Diagnosis of Salivary Gland Tumors through Inflammatory Biomarkers and Radiomics Metrics: A Preliminary Study

**DOI:** 10.3390/cancers15061876

**Published:** 2023-03-21

**Authors:** Umberto Committeri, Simona Barone, Giovanni Salzano, Antonio Arena, Gerardo Borriello, Francesco Giovacchini, Roberta Fusco, Luigi Angelo Vaira, Alfonso Scarpa, Vincenzo Abbate, Lorenzo Ugga, Pasquale Piombino, Franco Ionna, Luigi Califano, Giovanni Dell’Aversana Orabona

**Affiliations:** 1 Maxillofacial Surgery Operative Unit, Department of Neurosciences, Reproductive and Odontostomatological Sciences, Federico II University of Naples, 80131 Naples, Italy; 2 Department of Maxillo-Facial Medicine Surgery, Hospital of Perugia, 06132 Perugia, Italy; 3 Medical Oncology Division, Igea SpA, 80013 Naples, Italy; 4 Maxillofacial Surgery Operative Unit, Department of Medical, Surgical and Experimental Sciences, University of Sassari, 07100 Sassari, Italy; 5 Department of Medicine, Surgery and Dentistry, University of Salerno, 84084 Salerno, Italy; 6 Department of Advanced Biomedical Sciences, University of Naples “Federico II”, Via S. Pansini, 5, 80131 Naples, Italy; 7 Otolaryngology and Maxillo-Facial Surgery Unit, Istituto Nazionale Tumori—IRCCS Fondazione G. Pascale, 80131 Naples, Italy

**Keywords:** radiomics, machine learning, salivary gland tumors, systemic immune-inflammation index, platelet-to-lymphocyte ratio, neutrophil-to-lymphocyte ratio, systemic inflammation response index

## Abstract

**Simple Summary:**

The management of salivary gland tumors (SGTs), especially their early diagnosis, remains a challenge for physicians. Indeed, differentiating benign and malignant SGTs is an essential step in choosing an appropriate surgical approach. The aim of this study was to increase the effectiveness of pre-surgical diagnosis through a machine learning (ML) diagnostic tool that evaluates inflammatory biomarkers and radiomic metrics extracted from magnetic resonance imaging (MRI) sequences. Specifically, we considered the following indices of inflammation as inflammatory biomarkers: the systemic immune-inflammation index (SII), the systemic inflammation response index (SIRI), the platelet-to-lymphocyte ratio (PLR), and the neutrophil-to-lymphocyte ratio (NLR). In the context of cancer research, however, radiomics enables high-performance quantitative analysis of radiological images. We concluded that inflammatory biomarkers and radiomic features are comparably capable of supporting a differential diagnosis and are easily obtained through the preclinical investigations of patients.

**Abstract:**

Background: The purpose of this study was to investigate how the systemic inflammation response index (SIRI), systemic immune-inflammation index (SII), neutrophil/lymphocyte ratio (NLR) and platelet/lymphocyte ratio (PLR), and radiomic metrics (quantitative descriptors of image content) extracted from MRI sequences by machine learning increase the efficacy of proper presurgical differentiation between benign and malignant salivary gland tumors. Methods: A retrospective study of 117 patients with salivary gland tumors was conducted between January 2015 and November 2022. Univariate analyses with nonparametric tests and multivariate analyses with machine learning approaches were used. Results: Inflammatory biomarkers showed statistically significant differences (*p* < 0.05) in the Kruskal–Wallis test based on median values in discriminating Warthin tumors from pleomorphic adenoma and malignancies. The accuracy of NLR, PLR, SII, and SIRI was 0.88, 0.74, 0.76, and 0.83, respectively. Analysis of radiomic metrics to discriminate Warthin tumors from pleomorphic adenoma and malignancies showed statistically significant differences (*p* < 0.05) in nine radiomic features. The best multivariate analysis result was obtained from an SVM model with 86% accuracy, 68% sensitivity, and 91% specificity for six features. Conclusions: Inflammatory biomarkers and radiomic features can comparably support a pre-surgical differential diagnosis.

## 1. Introduction

The correct preoperative diagnosis and effective treatment of salivary gland tumors (SGTs) remain a challenge due to their heterogeneous histology, despite numerous recent advances in the literature. The current 2017 World Health Organization (WHO) classifications include 11 histologically benign tumors (54–79%), which are the most frequent types, particularly pleomorphic adenoma and Warthin tumors, and 24 malignant tumors (21–46%) [1]. The first steps in preoperative evaluation are ultrasonography (USG) and magnetic resonance imaging (MRI) to determine the location of the tumor and needle aspiration cytology (FNAC) to establish a diagnosis before surgical treatment. The sensitivity and specificity of FNAC range from 0.74 to 0.88 and 0.87 to 0.98, respectively, which are both higher for benign lesions and lower in cases of malignant tumors [2,3,4]. Inflammatory biomarkers could play a key role as a diagnostic support in cases of dubious cytology diagnosis in SGT, as has already been recently studied in the literature [5]. Among these prognostic factors, the best known are the neutrophil-to-lymphocyte ratio (NLR); platelet-to-lymphocyte ratio (PLR); systemic inflammation response index (SIRI), defined as the neutrophil count × monocyte/lymphocyte count; and systemic immune-inflammation index (SII), calculated with the formula neutrophils × platelets/lymphocytes. In addition, through the appropriate use of techniques from the areas of machine learning and artificial intelligence, the quantitative analysis of pathology-related images can be performed with high performance. Radiomics, in fact, obtains descriptors of image contents by extracting quantitative features from medical images [6]. The aim of this study was to increase the effectiveness of presurgical diagnosis and improve differentiation between benign and malignant SGT through the application of these new diagnostic tools, namely inflammatory biomarkers and radiomic metrics extracted from nuclear magnetic resonance (NMR) image sequences.

## 2. Materials and Methods

### 2.1. Patient Selection

As part of a retrospective study, 117 patients were recruited from the Maxillofacial Surgery Department of Policlinico Federico II in Naples. The study was conducted between January 2015 and November 2022. Of a total of 648 referrals reviewed, 117 patients were eligible for this study, meeting the following inclusion criteria: 

Patients had histological confirmation of malignant or benign SGT. Out of a total of 648 referrals reviewed, 117 patients were eligible with a preoperative salivary gland FNAC.

Complete medical records were available, including clinical and hematologic parameters. 

T1WI and T2WI sequences of MRI scans were complete, available, and free of artifacts.

Patients did not receive radiotherapy or chemotherapy treatments prior to the examination.

Since inflammatory biomarker levels may be influenced by other factors, patients whose medical histories included previous cancer at other sites, chronic inflammatory or autoimmune diseases, infections, serum viral markers, hematologic disorders, or concurrent or long-term anti-inflammatory or steroid drug treatments were excluded from this study.

This study was conducted in accordance with the Declaration of Helsinki, and because of its retrospective nature, local ethics committee approval was not required. 

Blood samples were collected for neutrophil, monocyte, lymphocyte, and platelet counts and measured in the laboratory 10 days before surgery. The NLR was calculated by dividing the absolute neutrophil count (N) by the absolute lymphocyte count (L). The PLR was calculated by dividing the absolute platelet count (P) by the absolute lymphocyte count (L). 

The IBS was calculated by multiplying the absolute platelet count (P) and the neutrophil count (N) and dividing by the absolute lymphocyte count (L) (IBS = P × N/L).

The SIRI was calculated by multiplying the absolute neutrophil count (N) and the monocyte count (M) and dividing the result by the absolute lymphocyte count (L) (SIRI = N × M/L).

### 2.2. MRI Protocol

All patients underwent MRI exams using either a 1.5T (Gyroscan Intera, Philips, Eindhoven, The Netherlands) or a 3T MRI scanner (Magnetom Trio, Siemens Medical Solutions, Erlangen, Germany). The imaging protocol always included a coronal T2-weighted (T2-w) image.

### 2.3. Image Processing

**Figure 1:**
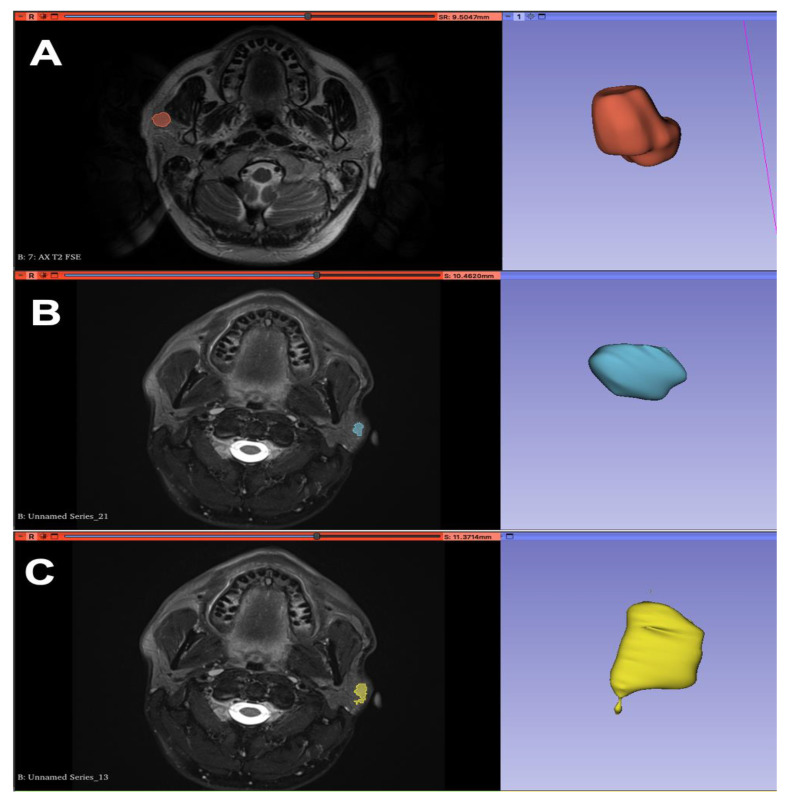
Segmentation of MRI images in axial T2 projection with 3D reconstruction of the ROI (region of interest) using 3D Slicer software: pleomorphic adenoma (**A**), Warthin tumor (**B**), malignant neoplasm (**C**).

Regions of interest (ROIs) were drawn manually, section by section, by two experienced radiologists with 22 and 15 years of experience in head and neck imaging, respectively. The radiologists first performed the selection of ROIs separately and then jointly. The manual definition of ROIs was determined using the 3D Slicer segmentation tool (Figure 1) (https://www.slicer.org, accessed on 17 March 2021). For the radiomic analysis, 851 features were extracted using PyRadiomics, in accordance with the feature definitions described by the Imaging Biomarker Standardization Initiative (IBSI) [7]. More precisely, the set of 851 radiomic features were extracted from the tumor region after manual segmentation. The radiomic features were divided into the following classes: first-order, shape-based statistics (2D and 3D); gray-level co-occurrence matrix (24 features); gray-level sequence length matrix (16 features); gray-level size area matrix; adjacent gray tone difference matrix; and gray-level dependence matrix. Details on radiomic features can be found in Kumarasamy’s work [8].

### 2.4. Statistical Analysis

#### 2.4.1. Univariate Analysis

The non-parametric Kruskal–Wallis test was performed to identify those features with potential to differentiate Warthin tumors from pleomorphic adenoma and malignant carcinoma on an individual basis. Receiver operating characteristic (ROC) analysis was also performed, and the Youden index was used to identify the optimal cut-off value for each feature. The area under the ROC curve (AUC), sensitivity, specificity, positive predictive value (PPV), negative predictive value (NPV), and accuracy were then calculated to evaluate the ability to differentiate benign lesions (Warthin tumors and pleomorphic adenoma) from malignant ones. The McNemar test was used to assess statistically significant differences between diagnostic performances.

The univariate analyses were performed using the Statistics Toolbox of MATLAB R2007a (MathWorks, Natick, MA, USA).

#### 2.4.2. Multivariate Analysis

A multivariate analysis was performed to discriminate benign lesions (Warthin tumors and pleomorphic adenoma) from malignant ones. Clinical parameters and radiomics features were considered in combination.

Given the high number of radiomics features, the selection of variables was performed based on the results obtained from the univariate analysis before proceeding with this multivariate analysis. This selection considered only the features that were significant in the Kruskal–Wallis test and the features that obtained an accuracy ≥ 65%.

The linear regression model was used to evaluate the best linear combination of significant characteristics for each outcome. In addition, pattern recognition methods including support vector machine (SVM), k-nearest neighbors (KNN), artificial neural network (NNET), and decision tree (DT) were adopted to evaluate performance in a multivariate procedure. The best multivariate model was chosen based on the highest accuracy. The dataset was randomly split into three independent sets in a 70:10:20 ratio for training, validation, and testing:

- Training Set: The dataset that we fed our model to learn potential underlying patterns and the relationships between them.
- Validation Set: The dataset that we used to understand our model’s performance across different model types and hyperparameter choices.
- Test Set: The dataset that we used to approximate our model’s unbiased accuracy in the wild.

MATLAB R2007 (MathWorks, Natick, MA, USA) was used for the statistical analysis and machine learning. A *p*-value < 0.05 was considered significant.

## 3. Results

A total of 47 patients with Warthin tumors, 42 patients with pleomorphic adenoma, and 28 patients with malignant neoplasms were enrolled and analyzed. The patient characteristics and the median values and ranges of the clinical parameters are reported in Table 1.

**Figure 2:**
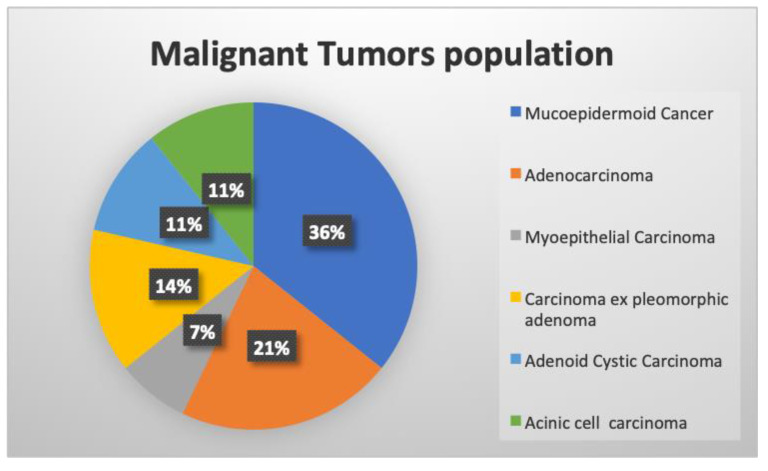
Main histopathological types of the malignant population studied.

Main histopathological types of malignant neoplasms and localization are reported in Figure 2. The 28 malignant tumors were low-grade, and all cytological diagnoses were confirmed by definitive histological results.

### 3.1. Univariate Analysis

**Figure 3:**
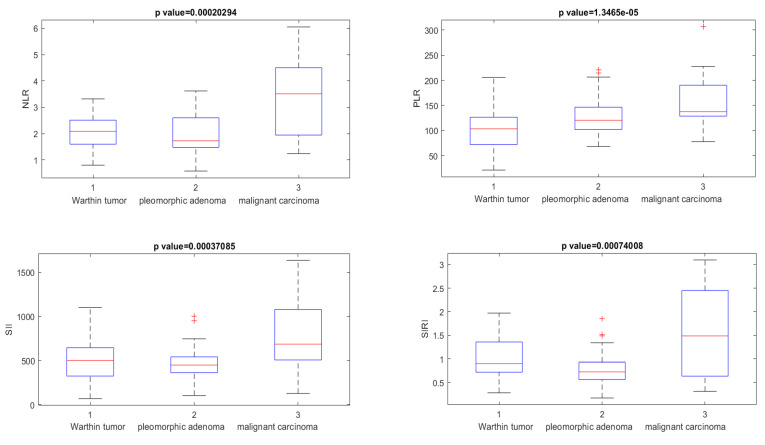
Boxplot of NLR, PLR, SII, and SIRI used to differentiate Warthin tumors from pleomorphic adenoma and malignant carcinoma. The + symbol represents the outliers.

All clinical parameters (NLR, PLR, SII, and SIRI) showed statistically significant (*p* < 0.05) differences in the Kruskal–Wallis test based on median values when discriminating Warthin tumors from pleomorphic adenoma and malignant tumors (Figure 3).

When discriminating Warthin tumors from pleomorphic adenoma and malignant SGT, the clinical parameters NLR, PLR, SII, and SIRI showed accuracies of 0.88, 0.74, 0.76, and 0.83, respectively, with appreciable values of the AUC (>0.70, see Table 2).

Univariate analysis of the radiomic characteristics showed nine statistically significant differences in the median values (Kruskal–Wallis, *p* < 0.05) when differentiating Warthin tumors from pleomorphic adenoma and malignant tumors. These nine characteristics are reported in Table 2 with their respective diagnostic performances.

The best result among both the clinical and radiomic parameters was seen in the ROC analysis for NLR, with an AUC of 0.74, a specificity of 1, a PPV of 1, and an NPV of 0.86. However, a low sensitivity value (sensitivity of 0.50) was observed.

Considering the selection of significant features from the univariate analysis, with an accuracy ≥ 70%, six features (two radiomics metrics and four clinical parameters) were used for the multivariate analysis: wavelet_LHL_gldm_LargeDependenceEmphasis, wavelet_HLH_glcm_JointEnergy, NLR, PLR, SII, and SIRI.

### 3.2. Multivariate Analysis

**Figure 4:**
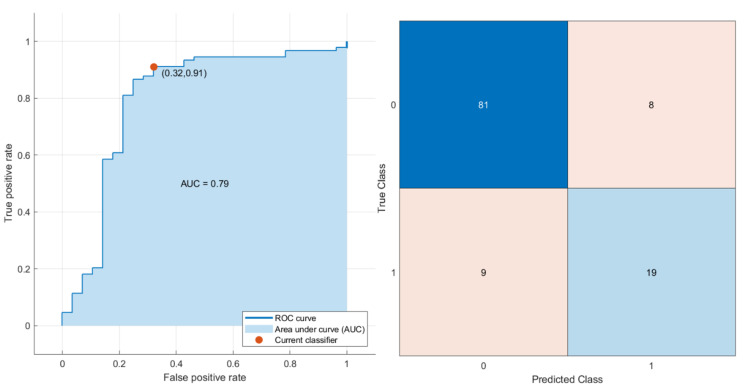
ROC curve and confusion matrix of the SVM model.

Considering the six significant features (wavelet_LHL_gldm_LargeDependenceEmphasis, wavelet_HLH_glcm_JointEnergy, NLR, PLR, SII, and SIRI), the best result in the multivariate analysis was achieved by an SVM model with an accuracy of 86%, a sensitivity of 68%, and a specificity of 91% (prediction speed: ~1500 obs/sec; training time: 4.2595 s; kernel function: cubic; hyperparameter options disabled). Figure 4 reports the ROC curve and the confusion matrix of the SVM model based on the test dataset.

However, there was no statistically significant difference between the accuracy of NLR considered alone compared to the accuracy of the SVM model trained with the six clinical and radiomics parameters (*p* value > 0.05 for the McNemar test).

## 4. Discussion

Considering that the management and early diagnosis of SGT remain a challenge for physicians, blood tests (inflammatory biomarkers) and MRI radiomic signatures (Rad-Score) could play an important role in the early diagnosis of the disease.

The information that these measures provide to physicians about the inflammatory process and discrimination between different tumor types can be combined with other patient characteristics, when available, to increase the power of decision support models.

In our study, we evaluated the potential of inflammatory biomarkers and radiomic features for the early diagnosis of SGT, both individually and in combination. Specifically, indices of inflammation (NLR, PLR, SII, and SIRI) were evaluated for their potential as inflammatory biomarkers. 

The roles of these inflammatory biomarkers have been extensively evaluated in the literature in various malignancies: breast cancer, colorectal cancer, renal cell carcinoma, non-small-cell lung cancer, esophageal squamous cell carcinoma, and salivary gland malignancies. Increased values of these inflammatory biomarkers before treatment were positively correlated with a poor prognosis [7,9,10,11,12,13,14]. 

Damar et al. were the first to establish that an elevated NLR could be used as an inflammatory marker to distinguish low-grade from high-grade parotid gland malignancies. In this study, NLR was significantly raised in malignant SGT compared to benign SGT (2.13 +/− 1.26 in benign and 3.29 +/− 3.13 in malignant) [15]. Guangyan Cheng et al. determined that an elevated NLR value before treatment is significantly associated with a poorer prognosis. In patients with an NLR < 2.48, the 10-year disease-specific survival (DSS) rate was 68%; in patients with an NLR ≥ 2.48, the 10-year disease-specific survival (DSS) rate was 58% [16]. 

In addition, the combination of these biomarkers, particularly the SII and SIRI scores, has been evaluated as useful in preoperative risk stratification to effectively guide the treatment strategy and postoperative follow-up of patients with salivary gland malignancies. This combination, consolidating all three parameters simultaneously in the formula, has the capacity to fully assess the balance between the host immune and inflammatory conditions [17]. Inflammatory biomarkers, in addition to playing a supportive role in the assessment of poor prognosis, can also be used as diagnostic tools in difficult cases to provide proper guidance for the treatment of these tumors. Abbate et al. demonstrated that there are statistically significant increases in NLR, PLR, and SII indices in malignant SGT compared with benign SGT, thus establishing a cut-off value that is useful in decision making in SGT management [5]. The analysis of our results revealed cut-off values for differentiating a benign lesion from a malignant one (PLR: 133.30; SII: 594.91; SIRI: 1.61; NLR: 3.62), with a higher accuracy for SIRI (0.83) and NLR (0.88).

Although the exact mechanism underlying the associations of inflammatory biomarkers with benign and malignant lesions remains unknown, previous results suggest some possible explanations.

These results provide some insight into the role of the inflammatory state in benign and malignant salivary pathologies and how it may support the genesis of these malignant tumors. In fact, the inflammatory cells, such as lymphocytes, neutrophils, and platelets, contribute to the invasion of cancer cells into the peripheral blood.

Neutrophils secrete large amounts of vascular endothelial growth factor, fostering an appropriate microenvironment for the promotion of local tumor invasion and metastasis, along with platelets, suppressing the effective immune response mediated by lymphocytes.

Another pre-surgical instrumental examination used to support diagnosis in SGT is nuclear magnetic resonance imaging (MRI).

MRI is often the preferred imaging modality for patients with SGT; in fact, compared with CT, it avoids emitting ionizing radiation to patients and can better map the extent of the disease with contrast resolution, especially in the depiction of local spread and the relationship of the tumor with the facial nerve and its branches [18]. Primarily, MRI-based tumor diagnosis by qualitative assessment relies on the radiologist’s experience, which can result in a less objective assessment, especially in a complex region such as the head or neck. For example, some features of benign tumors might mislead the observer if they are typically malignant features, such as those having irregular margins. Conversely, some low-grade malignant tumors might have benign features. Therefore, quantitative methods that can be used to differentiate between malignant and benign SGTs could improve diagnostic accuracy and reduce inter-observer variability. In this case, radiomic analysis of MRI radiomic features involves the extraction of quantitative imaging features with high throughput from images of the SGT region. Moreover, in the context of oncology studies, approaches based on artificial intelligence, machine learning, and radiomic metrics have been widely reported [19,20,21,22,23]. MRI radiomic signatures have also been recognized as preoperative and independent prognostic factors for head and neck squamous cell carcinoma (HNSCC) and nasopharyngeal carcinoma (NPC) in clinical practice [24,25]. In addition, Le-le Song et al. utilized a radiomics model that can provide objective and quantitative information on intra-tumor heterogeneity and inter-tumor microenvironments hidden in the image [26]. Rongli Zhang et al. studied the impact of reducing the number of initial radiomic features on the performance of radiomic models to differentiate between benign and malignant SGTs. They applied six feature categories separately and all the feature categories in combination from three anatomy-based MRI sequences [27].

This differentiation between benign and malignant SGTs is an essential step to ensure that a suitable surgical approach is taken. To date, the conventional methods used for pre-surgical differential diagnosis of major SGTs are ultrasonography, MRI, and FNAC, with sensitivities and specificities ranging from 0.74 to 0.88 and 0.87 to 0.98, respectively. FNAC accurately discerns the neoplastic nature of the lesion in most cases. However, the exact categorization of tumors, especially malignancies, can be challenging due to their cytomorphological heterogeneity. This leads to an often undiagnostic or indeterminate cytological diagnosis. In fact, the precision of FNAC for SGT subtyping is lower and varies from 62% to 80% [28,29,30]. For this reason, given the low accuracy of FNAC in certain cases, and given the growing use of inflammatory biomarkers and radiomic characteristics to help clinicians in the pre-surgical phase of many tumors, we were prompted to ascertain their validity, even for a group of tumors as heterogenous as SGTs.

In our study, the inflammatory biomarkers NLR, PLR, SII, and SIRI showed an accuracy of 0.88, 0.74, 0.76, and 0.83, respectively, in differentiating Warthin tumors from pleomorphic adenoma and malignant neoplasms. In addition, in the Kruskal–Wallis test based on median values for differentiating Warthin tumors from pleomorphic adenoma and malignant neoplasms, statistically significant differences (*p* < 0.05) were shown in nine radiometric features. The best result in the multivariate analysis was obtained from an SVM model with 86% accuracy, 68% sensitivity, and 91% specificity for six features.

However, this is a preliminary study that has some limitations. It is a retrospective study of 117 patients with SGTs, ROI segmentation was performed manually, and unknown or unreported inflammatory diseases could have influenced the results regarding the inflammatory biomarkers.

Additionally, the results may have been influenced by the small sample size. Future studies should aim to overcome these limitations.

## 5. Conclusions

In conclusion, the inflammatory biomarkers and radiological features identified with machine learning almost overlap, greatly supporting pre-surgical differential diagnosis. The main advantage of these methods is the ease with which the results are obtained through the patients’ preclinical investigations: blood tests and MRI. The calculation of inflammatory biomarkers may be easier than obtaining radiomic features. However, it is possible to obtain radiomic features through open-source software.

In any case, it must be considered that the use of inflammatory biomarkers as support tools is not suitable for all types of patients. Inflammatory biomarkers may be influenced by other factors such as chronic inflammation, autoimmune diseases, infections, hematologic disorders, and concurrent or long-term anti-inflammatory or steroid drug treatments.

## Figures and Tables

**Table 1:** Patient characteristics and median values and ranges of clinical parameters.

		Warthin Tumor	Pleomorphic Adenoma	Malignant Carcinoma
Gender	Male	32	15	16
	Female	15	27	12
Age	Median value	62	52.5	53.5
	Minimum	13	8	22
	Maximum	76	83	84
NLR	Median value	2.09	1.73	4.2
	Minimum	0.8	0.58	1.56
	Maximum	3.32	3.62	6.05
PLR	Median value	103.64	120.69	148.63
	Minimum	21.67	68.56	78
	Maximum	205.46	220.2	306.14
SII	Median value	502.03	451.17	932.04
	Minimum	71.32	105.7	129.49
	Maximum	1103.36	1005.4	1634.4
SIRI	Median value	0.9	0.725	1.73
	Minimum	0.28	0.17	0.31
	Maximum	1.97	1.85	3.1

**Table 2:** Diagnostic performance of clinical and radiomic parameters based on univariate analysis.

Radiomics Metric	AUC	Sensitivity	Specificity	PPV	NPV	Accuracy	Cut-Off
wavelet_HHL_glcm_ClusterTendency	0.34	1.00	0.01	0.24	1.00	0.25	0.06
wavelet_HHL_glszm_ LargeAreaHighGrayLevelEmphasis	0.65	0.86	0.45	0.33	0.91	0.55	0.58
wavelet_HHL_glszm_ LargeAreaLowGrayLevelEmphasis	0.67	0.93	0.44	0.34	0.95	0.56	0.33
wavelet_LLH_gldm_ LargeDependenceLowGrayLevelEmphasis	0.64	0.89	0.47	0.35	0.93	0.57	−5.61
wavelet_LLH_gldm_ LargeDependenceEmphasis	0.64	0.79	0.56	0.36	0.89	0.62	−5.06
wavelet_HHL_glcm_ ClusterProminence	0.36	0.29	0.74	0.26	0.77	0.63	0.28
wavelet_LHL_gldm_ LargeDependenceLowGrayLevelEmphasis	0.67	0.79	0.63	0.40	0.90	0.67	−2.43
wavelet_LHL_gldm_ LargeDependenceEmphasis	0.68	0.75	0.69	0.43	0.90	0.70	−2.24
wavelet_HLH_glcm_JointEnergy	0.61	0.50	0.78	0.41	0.83	0.71	0.79
PLR	0.74	0.71	0.75	0.48	0.89	0.74	133.30
SII	0.73	0.71	0.78	0.50	0.90	0.76	594.91
SIRI	0.68	0.50	0.93	0.70	0.86	0.83	1.61
NLR	0.74	0.50	1.00	1.00	0.86	0.88	3.62

## Data Availability

Not applicable.
